# Environmental Enrichment Promotes Transgenerational Programming of Uterine Inflammatory and Stress Markers Comparable to Gestational Chronic Variable Stress

**DOI:** 10.3390/ijms24043734

**Published:** 2023-02-13

**Authors:** Nayara A. Lopes, Mirela Ambeskovic, Stephanie E. King, Jamshid Faraji, Nasrin Soltanpour, Erin A. Falkenberg, Taylor Scheidl, Mansi Patel, Xin Fang, Gerlinde A. S. Metz, David M. Olson

**Affiliations:** 1 Department of Obstetrics and Gynecology, University of Alberta, Edmonton, AB T6G 2R3, Canada; 2 Department of Physiology, University of Alberta, Edmonton, AB T6G 2R3, Canada; 3 Department of Neuroscience, University of Lethbridge, Lethbridge, AB T1K 3M4, Canada; 4 Department of Pediatrics, University of Alberta, Edmonton, AB T6G 2R3, Canada

**Keywords:** chronic variable stress, pregnancy, uterus, prenatal stress, preterm birth, gene expression, inflammation, rodents, enriched environment, resilience

## Abstract

Prenatal maternal stress is linked to adverse pregnancy and infant outcomes, including shortened gestation lengths, low birth weights, cardio-metabolic dysfunction, and cognitive and behavioural problems. Stress disrupts the homeostatic milieu of pregnancy by altering inflammatory and neuroendocrine mediators. These stress-induced phenotypic changes can be passed on to the offspring epigenetically. We investigated the effects of gestational chronic variable stress (CVS) in rats using restraint and social isolation stress in the parental F0 generation and its transgenerational transmission across three generations of female offspring (F1–F3). A subset of F1 rats was housed in an enriched environment (EE) to mitigate the adverse effects of CVS. We found that CVS is transmitted across generations and induces inflammatory changes in the uterus. CVS did not alter any gestational lengths or birth weights. However, inflammatory and endocrine markers changed in the uterine tissues of stressed mothers and their offspring, suggesting that stress is transgenerationally transmitted. The F2 offspring reared in EE had increased birth weights, but their uterine gene expression patterns remained comparable to those of stressed animals. Thus, ancestral CVS induced changes transgenerationally in fetal programming of uterine stress markers over three generations of offspring, and EE housing did not mitigate these effects.

## 1. Introduction

Evidence from animal and human studies suggests that prenatal maternal stress (PNMS) is associated with preterm birth, preeclampsia, and adverse lifelong health outcomes in the offspring [1,2,3,4,5,6]. Adversity during critical periods of embryonic and fetal development may lead to modifications of the transcriptome that alter gene expression and phenotypes in adulthood [7,8]. This concept is known as the “Barker hypothesis” [9,10]. However, the PNMS mechanisms underlying the adverse pregnancy outcomes (APOs) and fetal programming are not well understood.

When a pregnant female experiences adversity, the impacts of that stress affect the exposed somatic tissues (filial (F0 generation), the fetuses (F1 generation), and the fetuses’ germline (F2 generation) [11]. A true transgenerational inheritance arises when the phenotypic changes observed in the stressed individuals persist in the unexposed F3 offspring. This occurs through germline epimutations that are transmitted to the descendants [11,12].

Maternal neuroendocrine responses to stress and immune function must adapt significantly during pregnancy to support fetal growth and development [13], characterizing a vulnerable time to stress. The hypothalamic–pituitary–adrenal (HPA) axis and the immune system are two of the most relevant systems affected by gestational stress [14]. Several studies have shown a link between prenatal stressors and altered endocrine and inflammatory mediators in the gestational tissues [3,5,15,16,17]. Excess maternal cortisol/corticosterone (CORT) is released during stress and may cross the placenta, directly affecting the fetal brain and HPA axis [18]. This elevated CORT concentration surpasses the protective actions of 11β-hydroxysteroid dehydrogenase type 2 (11β-HSD2), which converts active glucocorticoids (GCs) into inactive ketone products [19]. Therefore, stress disrupts pregnancy homeostasis and may lead to adverse maternal and child outcomes.

During the inflammatory process of labour, cytokine and chemokines are amplified and modulate coordinated physiological processes that promote uterine transitioning into an active state [20,21]. When exposed to acute and chronic stressors, pregnant women exhibited higher plasma concentrations of proinflammatory cytokines such as interleukin (IL)-1β, IL-6 (IL-6), and tumour necrosis factor alpha (TNF-α) [22,23,24] and lower levels of the anti-inflammatory cytokine IL-10 (IL-10) [22]. Further studies demonstrated that changes in the immune system and cytokine milieu during PNMS are associated with poor pregnancy outcomes, including preterm birth [4,25,26,27]. Similarly, prenatal exposure to social isolation and restraint stress in rats induced changes in the uterine expression of inflammatory markers [17,28].

Maternal stress is also associated with cellular redox dysregulation [29,30,31,32], which contributes to poor outcomes such as neuropsychiatric disorders [30] and preterm birth [33]. Both proinflammatory cytokines [34,35] and hypoxia [36,37] downregulate placental 11β-HSD2, potentially leading to excessive fetal exposure to maternal CORT. Therefore, the stress-induced impaired coordination of the HPA-immune system and reactive oxygen species (ROS) production in mothers may, at least in part, explain the early programming of diseases [38].

Strategies to reduce the effects of stress include healthy environments and lifestyle choices that promote physical and mental benefits to lifelong human health. In animals, the enriched environment (EE) intervention has become considered to be a stress-reduction technique that improves the physical and social environment [39,40]. EE promotes social interaction by housing more animals in larger cages. Animals are also provided with various toys with diverse textures, colours, and shapes to encourage cognitive, sensory, and motor stimulation [39,41].

Preconceptional and gestational EE housing contributed to pregnancy maintenance and reduced preterm birth rates by 40% in an inflammatory mouse model by dampening the inflammatory response and pro-labour mediators [42]. The EE intervention also improved behavioural, morphological, and molecular parameters resulting from the adverse programming of ancestral stress [43,44]. Yet, little is known about the efficacy of EE housing on improving transgenerational uterine programmed effects of PNMS and stress-induced preterm birth risk.

**Figure 1:**
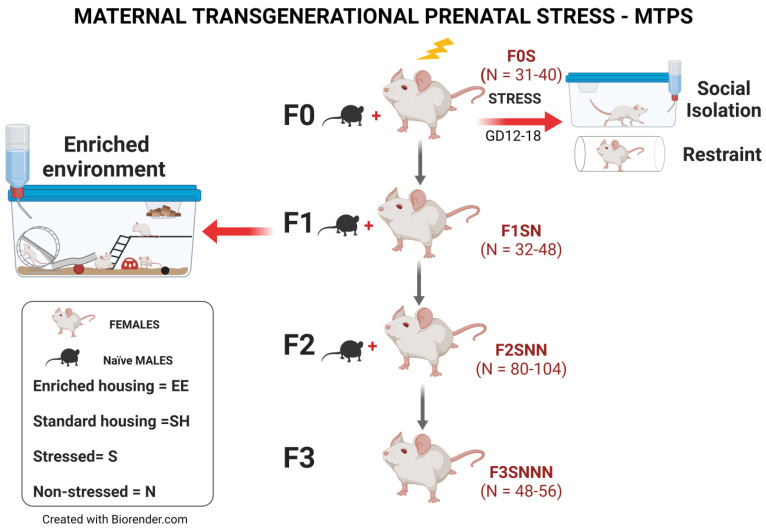
Maternal transgenerational prenatal stress experimental design. Flow chart illustrating that F0 pregnant dams were subjected to social isolation and restraint stress from GD12–18. EE housing included a combination of physical and sensorimotor enrichment and was implemented only in the F1 generation from weaning to GD20. The F1 (SN–EE) dams were then bred with nonstressed males to assess whether EE mitigated the negative effects of PNMS, and if these mitigative effects would be passed down to their F2 and F3 offspring. Therefore, the F2 and F3 generations were not directly exposed to EE housing or stress but experienced their transgenerational ancestral effects. F = filial generation. Created with Biorender.com (accessed on 15 July 2022).

The present study investigated the transgenerational effects of prenatal psychological and psychosocial stress over four generations on (1) offspring birth weights, (2) uterine expression of inflammatory and stress markers, and (3) preterm birth risk. Only the parental F0 generation was subjected to social isolation stress and restraint, whereas the F1–F3 generations were left unstressed (Figure 1). We also assessed (4) whether housing F1 daughters exposed to ancestral stress under EE housing would improve stress-induced adverse outcomes in their uteri and in future generations. We hypothesized that PNMS induces uterine molecular changes of inflammatory and stress markers and leads to preterm birth in a transgenerational fashion. We also postulated that the transgenerational effects of adversity would be mitigated by enrichment in the progeny.

## 2. Results

### 2.1. Gestational Lengths Were Unchanged in the Stress Groups across Two Generations

**Figure 2:**
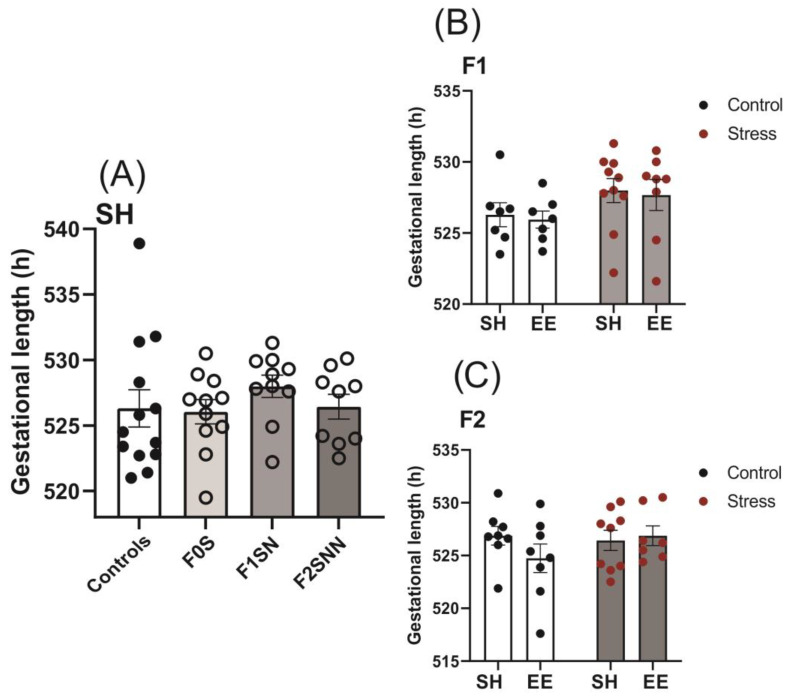
Gestational lengths were unchanged in animals subjected to PNMS. (**A**) Gestational lengths among treatment groups. Gestational lengths in control and stress groups exposed to either SH or EE housing conditions in the (**B**) F1 and (**C**) F2 generations. Data are compared to F0N, mean ± SEM. Ordinary one-way analysis of variance (ANOVA) (**A**) and two-way ANOVA (**B**,**C**) analyses were used. N = 9–13 (**A**) or 7–10 (**B**,**C**). SH = standard housing; EE = enriched housing; F = filial generation; S = stressed; N = nonstressed.

The gestational lengths for the F0–F2 stress groups remained unchanged compared to the controls (F0N 526.3 ± 5.12 h, F0S 526 ± 3.05 h, F1SN 528 ± 2.67 h, F2SNN 526.4 ± 2.85 h; F (3,39) = 0.5783, *p* = 0.633; Figure 2A). We did not find any statistically significant interactions between treatment and housing for the F1 and F2 generations (F (1,28) = 0.0002, *p* = 0.988; F (1,28) = 1.481, *p* = 0.234, respectively); the implementation of the EE intervention did not modify gestational lengths in the F1 and F2 generations compared to standard housing (SH) conditions (*p* > 0.05; Figure 2B,C).

### 2.2. Offspring Birth Weights Were Unchanged in the Stress Group but Were Increased with Environmental Enrichment

**Figure 3:**
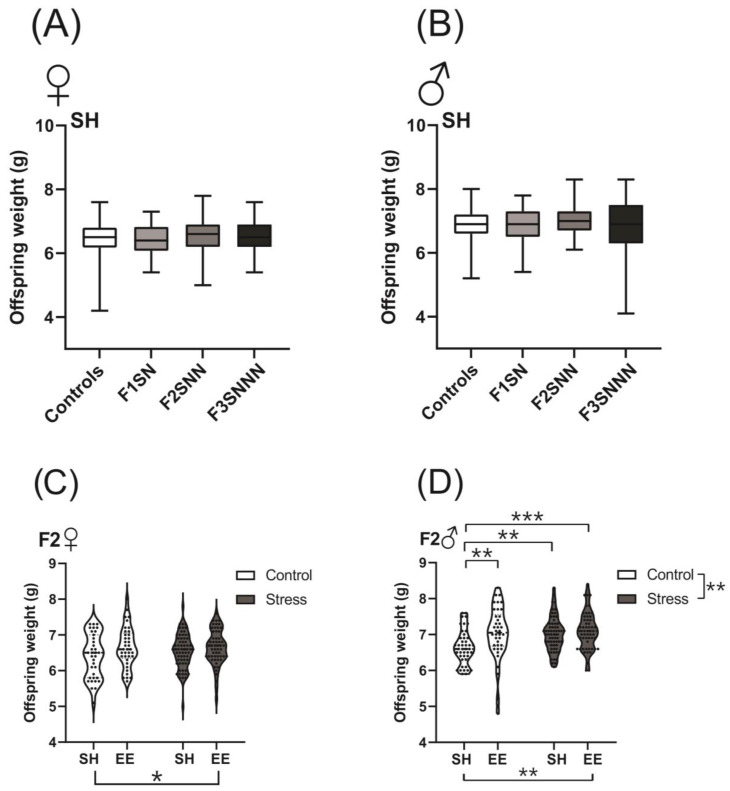
Offspring weights remained unchanged between treatment groups, whereas EE housing significantly increased neonatal birth weights of females and males in the F2 generation. Offspring weights between treatment groups in (**A**) females and **(B**) males. Pup weights of control and stressed (**C**) females and (**D**) males subjected to SH or EE housing. Data are compared to F1NN (**A**,**B**) and F2NNN (**C**,**D**), mean ± SEM. Box plot mid-lines indicate medians, whiskers indicate min-max values, and boxes indicate interquartile ranges. Kruskal-Wallis test (**A**,**B**) or two-way ANOVA (**C**,**D**) analyses were used. SH = standard housing; EE = enriched housing; F = filial generation; S = stressed; N = nonstressed. N = 35–65 (females, **A**,**C**); 38–66 (males, **B**,**D**). Asterisks indicate significance: <0.05 (*); <0.002 (**); <0.001 (***).

Stress has known adverse effects on neonatal birth weights [3,17,28]. We measured female and male weights on postnatal day (P)1 to determine if a combination of psychological and psychosocial stressors impacted offspring weight. Birth weights were unchanged in both the female (F1NN 6.45 g ± 0.52, F1SN 6.43 ± 0.51, F2SNN 6.54 ± 0.48, F3SNNN 6.51 ± 0.52; *p* = 0.673; Figure 3A) and male (F1NN 6.89 g ± 0.5, F1SN 6.84 ± 0.55, F2SNN 7.01 ± 0.48, F3SNNN 6.82 ± 0.82; *p* = 0.575; Figure 3) offspring between the control and stress groups.

We also tested the effects of enriched housing on neonatal birth weight. There was a significant effect of housing on P1 weights of female (F (1,188) = 5.239, *p* = 0.023; Figure 3C) and male neonates in the F2 generation (F2NNN SH vs. F2SNN *p* = 0.005; F2NNN SH vs. F2NNN EE *p* = 0.008; F2NNN SH vs. F2SNN EE *p* = 0.0004; overall ANOVA F (1,193) = 9.135, *p* = 0.003; Figure 3D). No interaction or treatment effects were observed in the F2 generation of female rats (F (1,188) = 1.053, *p* = 0.306 and F (1,188) = 0.741, *p* = 0.390, respectively; Figure 3C). Despite significant main effects of treatment in F2 male pups (F (1,193) = 7.029, *p* = 0.009), no interaction was observed (F (1,193) = 3.655, *p* = 0.057; Figure 3D).

**Figure 4:**
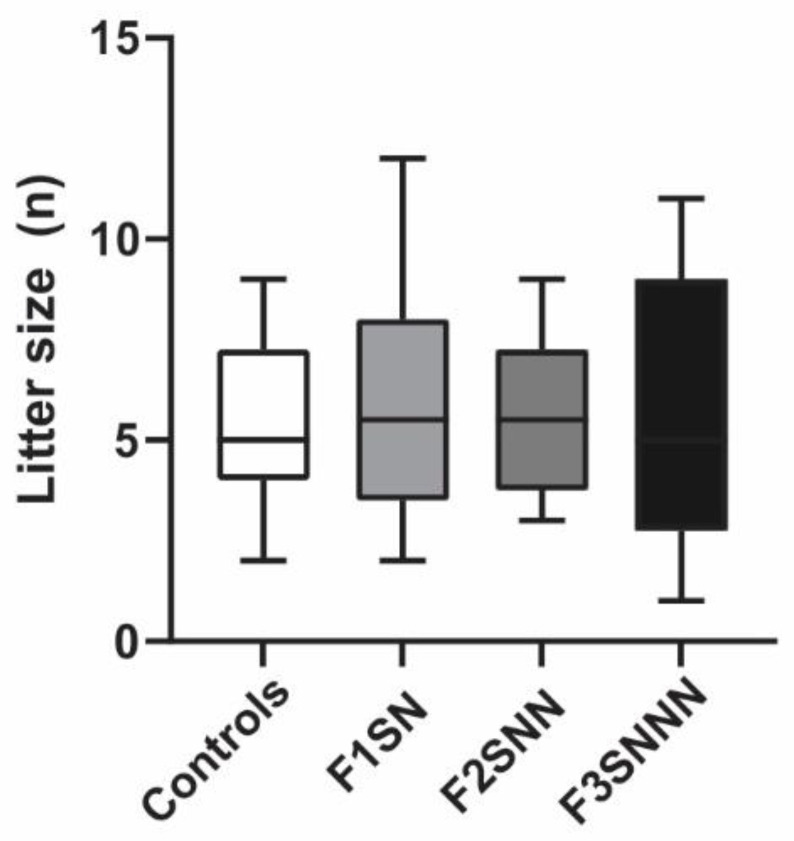
Litter sizes were unchanged between treatments. Data are compared to F1NN and were analyzed using the Kruskal-Wallis test. Box plot mid-lines indicate medians, whiskers indicate min-max values, and boxes indicate interquartile ranges. N = 14–22 per group. F = filial generation; S = stressed; N = nonstressed.

Two-way ANOVA revealed no differences in offspring birth weights between treatment and housing types for both F3 females and males (Supplementary Figure S1A,B). Furthermore, the litter sizes of the treatment groups remained unchanged (*p* = 0.983; Figure 4).

### 2.3. Concentrations of Plasma CORT Levels Were Increased in F1 and F2 Stressed Animals and Were Not Mitigated by EE Intervention

**Figure 5:**
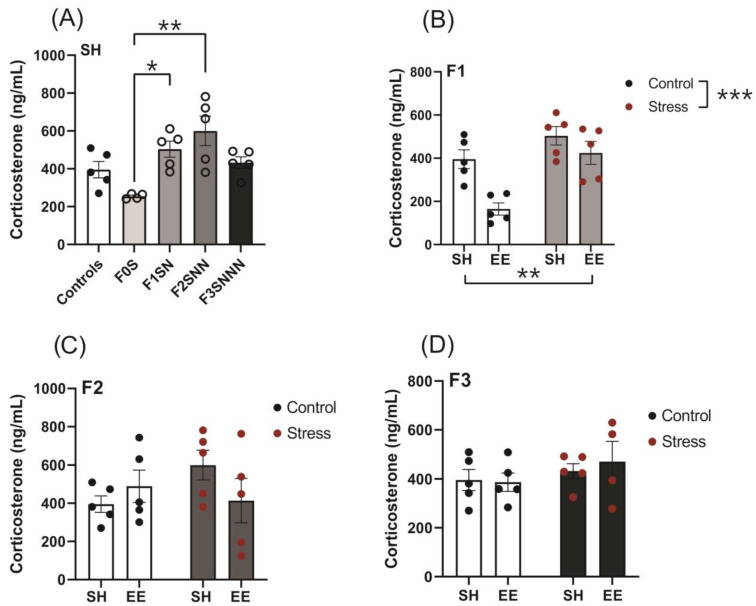
Elevated CORT concentrations in the F1 and F2 stressed offspring despite enrichment therapy. (**A**) Plasma CORT levels in the F0–F3 stressed and control animals. Effects of housing and treatment on the (**B**) F1, (**C**) F2, and (**D**) F3 generations. Asterisks indicate significance: <0.05 (*); <0.002 (**); <0.001 (***). Data are compared to F0N, mean ± SEM. Ordinary one-way ANOVA (**A**) and two-way ANOVA (**B**–**D**) analyses were used. N = 4–5 per group. SH = standard housing; EE = enriched housing; F = filial generation; S = stressed; N = nonstressed.

We measured CORT levels in plasma collected on gestational day (GD)18 (stressed dams) and P110 (adult offspring) to assess HPA axis activation and the release of GCs. CORT levels increased in the F1SN and F2SNN offspring (F (4,19) = 6.534, *p* = 0.002) when compared to F0S animals (*p* = 0.021, *p* = 0.001, respectively; Figure 5A). Two-way ANOVA revealed no significant interaction between the effects of stress and housing type on CORT levels in the F1–F3 offspring (F (1,16) = 3.105, *p* = 0.09; F (1,16) = 2.728, *p* = 0.118; F (1,15) = 0.2423, *p* = 0.630, respectively; Figure 5B–D). However, main effect analysis in the F1 generation showed a statistically significant effect of treatment and housing on CORT concentrations (F (1,16) = 18.40, *p* < 0.001; F (1,16) = 13.10, *p* = 0.002, respectively; Figure 5B).

### 2.4. Uterine ROS Levels Were Unchanged in Animals Exposed to Gestational and Ancestral Stress

Dihydroethidium (DHE) staining is widely used to assess the intracellular formation of ROS (originally designed to detect superoxide). DHE is a cell-permeable fluorescent dye that reacts with intracellular and extracellular superoxide to produce ethidium bromide, which binds to nuclear DNA and generates red fluorescence (excitation/emission wavelengths of 518/605 nm) [45]. We tested the effects of gestational (F0 generation) and ancestral (F1–F3 generations) stress on the formation of ROS in the uteri of exposed dams and evaluated whether EE housing would mitigate these effects.

**Figure 6:**
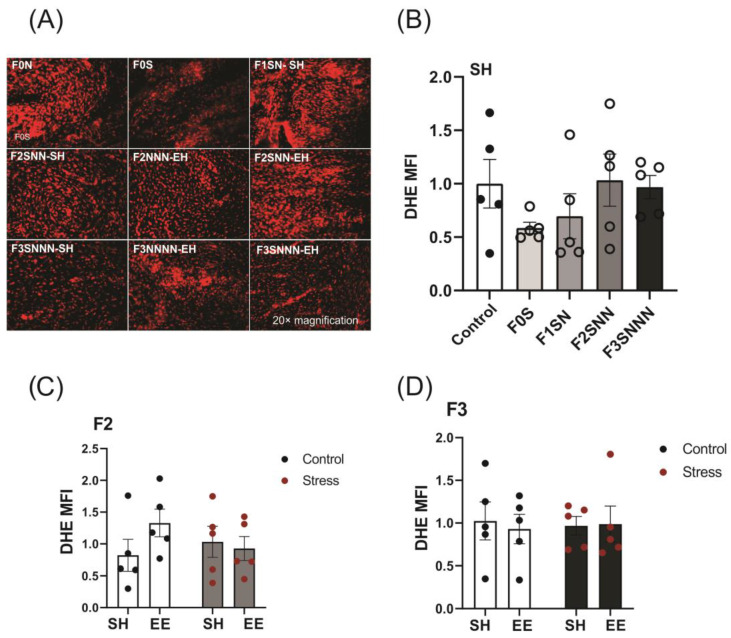
Uterine ROS levels were unchanged between treatments and housing conditions across the F0–F3 generations. Four random regions of each uterus were used to measure mean fluorescence intensity (MFI) with DHE staining. (**A**) Representative images for each treatment and housing group. (**B**) Analysis of ROS levels (mean fluorescence intensity, MFI) in uterine samples from stressed dams compared to controls. Assessments of the effects of EE housing on uterine ROS levels in the (**C**) F2 and (**D**) F3 generations of animals subjected to ancestral stress. Data are compared to F0N, mean ± SEM. Ordinary one-way ANOVA (**B**) and two-way ANOVA (**C**,**D**) analyses were used. N = 5. SH = standard housing; EE = enriched housing; F = filial generation; S = stressed; N = nonstressed.

Evaluation of ROS levels revealed unchanged levels between the control and stress groups over the F0–F3 generations (F (4,20) = 1.201, *p* = 0.341; Figure 6B). We did not observe a significant interaction between the effects of treatment and housing in either the F2 (F (1,16) = 1.830, *p* = 0.195; Figure 6C) or F3 generations (F (1,16) = 0.095, *p* = 0.762; Figure 6D). Two-way ANOVA showed insignificant effects of treatment and housing in the F2 and F3 offspring ROS levels (*p* > 0.05; Figure 6C,D).

### 2.5. Uterine Expression of Candidate Genes and Proteins Involved in Local Inflammatory Responses Were Impacted in Rats Exposed to Ancestral Stress

#### 2.5.1. Proinflammatory Cytokines: *Il1a* and *Il1b*

**Figure 7:**
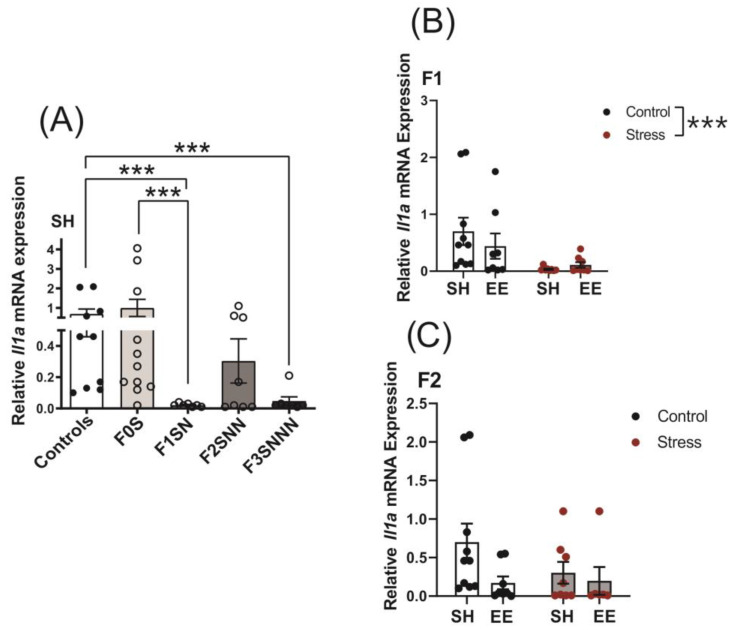
Uterine expression of *Il1a* was significantly downregulated in the F3 generation of stressed dams raised in SH and in F1 stressed animals exposed to both housing conditions. (**A**) Gene expression of *Il1a* in uteri of control and stressed dams across the F0–F3 generations. Uterine expression of *Il1a* in (**B**) F1 and (**C**) F2 animals exposed to different treatments and housing conditions. Asterisks indicate significance: <0.001 (***). Data are compared to F0N, mean ± SEM. Ordinary one-way ANOVA (**A**) and two-way ANOVA (**B**,**C**) analyses were used. N = 7–11 (**A**); 8–10 (**B**); or 6–10 (**C**). SH = standard housing; EE = enriched housing; F = filial generation; S = stressed; N = nonstressed.

Stress has been shown to alter proinflammatory cytokine expression in the uterus and levels in the blood [17,22,28,46]. Since proinflammatory cytokines are essential mediators of the inflammatory events leading to the activation of the birth cascade [21], we measured *Il1a* and *Il1b* gene expression in the uteri of rats that experienced gestational and ancestral stress. Uterine mRNA expression of *Il1a* decreased in the stressed F1 and F3 generations (F(4,38) = 8.774, *p* < 0.001; Figure 7A) compared to the controls (F0N vs. F1SN *p* < 0.001; F0N vs. F3SNNN *p* < 0.001) and F0S (F0S vs. F1SN *p* < 0.001).

We observed a main effect of treatment on *Il1a* expression in the F1 generation (F (1,30) = 18.180, *p* < 0.001; Figure 7B), but there was no significant interaction or main effect of housing (F (1,30) = 3.477, *p* = 0.072; F (1,30) = 0.237, *p* = 0.630, respectively). In addition, there was no significant interaction between treatment and housing on *Il1a* mRNA expression in the F2 generation (F (1,28) = 1.259, *p* = 0.271; Figure 7C). Two-way ANOVA revealed no significant interaction or main effects of treatment and housing on F3 uterine *Il1a* gene expression (Supplementary Figure S2A).

**Figure 8:**
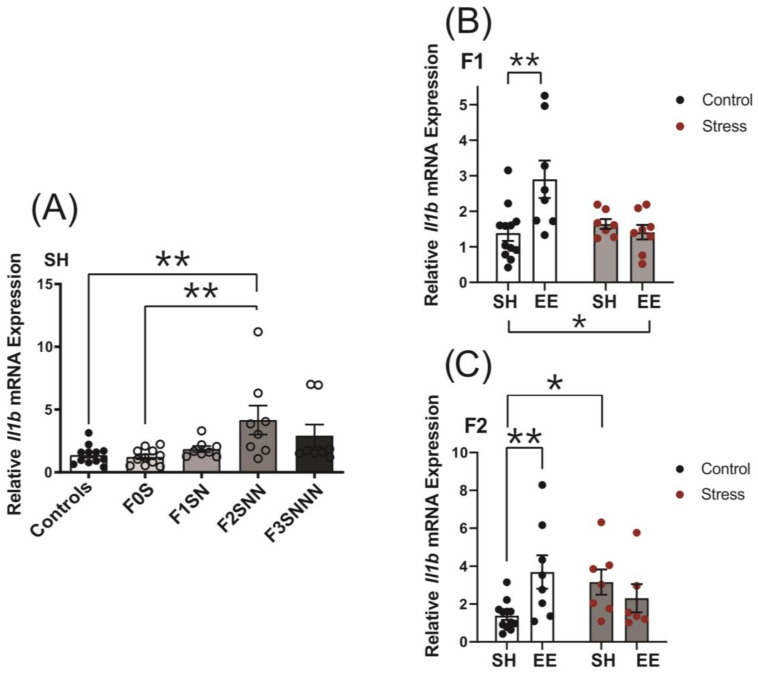
Uterine mRNA expression of *Il1b* increased significantly in the F2 generation exposed to transgenerational stress and in the F1 and F2 controls exposed to EE. (**A**) *Il1b* expression in F0–F3 dams subjected to CVS under SH. Uterine gene expression of *Il1b* in the (**B**) F1 and (**C**) F2 generations of controls and stressed animals exposed to SH or EE housing. Asterisks indicate significance: <0.05 (*); <0.002 (**). Data are compared to F0N, mean ± SEM. Ordinary one-way ANOVA (**A**) and two-way ANOVA (**B**,**C**) analyses were used. N = 8–12 (**A**); 7–12 (B); or 6–12 (**C**). SH = standard housing; EE = enriched housing; F = filial generation; S = stressed; N = nonstressed.

The expression of *Il1b* increased significantly in the F2 generation (F(4,42) = 5.491, *p* = 0.001; Figure 8A) compared to F0N and F0S (*p* < 0.002 for both). A statistically significant interaction between treatment and housing (F(1,31) = 7.915, *p* = 0.008; Figure 8B) and a significant main effect of housing (F(1,31) = 4.239, *p* = 0.048) were revealed in the *Il1b* expression analysis of the F1 generation. Pairwise comparisons showed a significant increase in *Il1b* expression in the F1NN-EE group compared to the F0N-SH controls (*p* = 0.002; Figure 8B). There was a significant interaction between treatment and housing for *Il1b* expression in the F2 generation (F (1,29) = 8.244, *p* = 0.008; Figure 8C), whereby *Il1b* significantly increased in the F2NNN-EE and F2SNN-SH groups compared to the F0N-SH controls (*p* = 0.007 and *p* = 0.019, respectively). Two-way ANOVA revealed no significant interaction or main effects of treatment and housing on *Il1b* mRNA expression in the F3 uteri (Supplementary Figure S2B).

Uterine mRNA expression of *Il6* and *Il1ra* demonstrated no significant differences between control and stressed animals across generations (Supplementary Figure S3A,B).

#### 2.5.2. Psychological and Psychosocial Stress in Tandem Did Not Induce Changes in IL-1RAP Protein Levels in Rat Uteri

**Figure 9:**
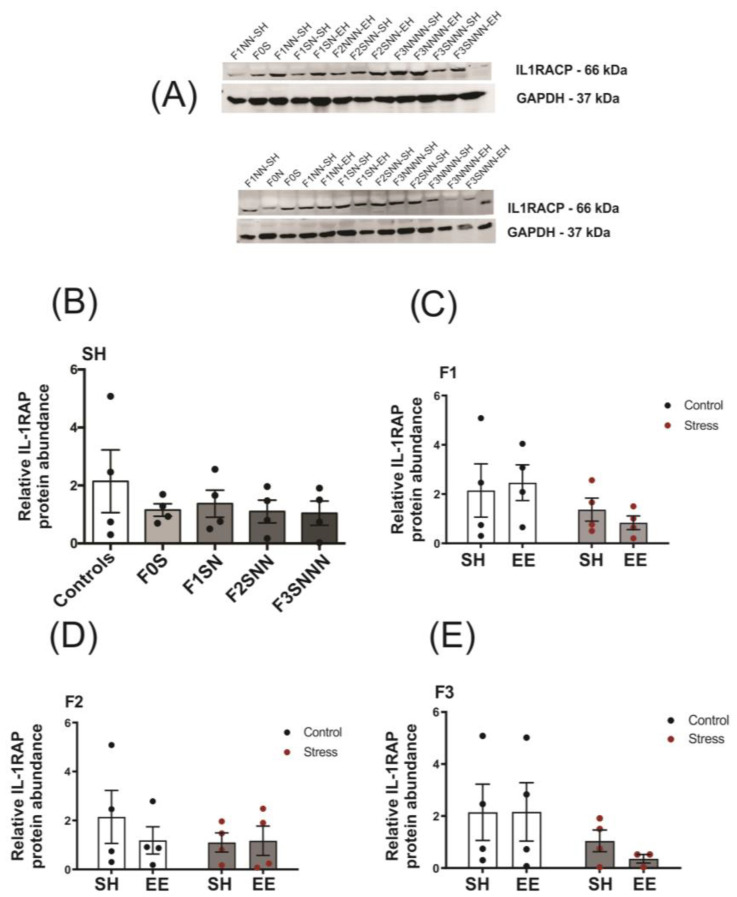
Uterine protein abundance of IL-1RAP remained unchanged over the stressed F0–F3 generations regardless of housing type. (**A**) IL-1RAP protein abundance quantified using densitometry (representative blots included). (**B**) Uterine protein abundance of IL-1RAP in F0–F3 stressed dams compared to controls. (**C**–**E**) IL-1RAP abundance according to treatment and housing across the F1–F3 offspring. Blots were quantitated using Odyssey software. All groups were compared using the Kruskal-Wallis test (**B**), and the effects of treatment and housing were assessed using two-way ANOVA (**C**–**E**). Data are normalized to glyceraldehyde 3-phosphate dehydrogenase (GAPDH) and compared to the F0N, mean ± SEM. N = 4 (**B**) or 3–4 (**C**–**E**). SH = standard housing; EE = enriched housing; F = filial generation; S = stressed; N = nonstressed.

The IL-1 receptor accessory protein (IL-1RAP) protein forms a complex with the IL-1 receptor 1 (IL-1R1) and, upon binding of IL-1 agonists, initiates downstream signalling events to active IL-1 responsive genes [47]. Protein abundance of IL-1RAP remained unchanged across the stress-exposed F0–F3 generations, as shown by Western blot analysis (*p* = 0.970; Figure 9B). Furthermore, we did not observe a significant interaction or main effects of treatment and housing types on IL-1RAP abundance in the uterus of F1–F3 adult offspring (*p* > 0.05; Figure 9C–E, respectively).

### 2.6. Uterine Expression of Candidate Genes Involved in Local Stress Responses Were Impacted in Rats Exposed to Ancestral Stress

#### 2.6.1. 11β-Hydroxysteroid Dehydrogenase Type 2 (*Hsd11b2*)

**Figure 10:**
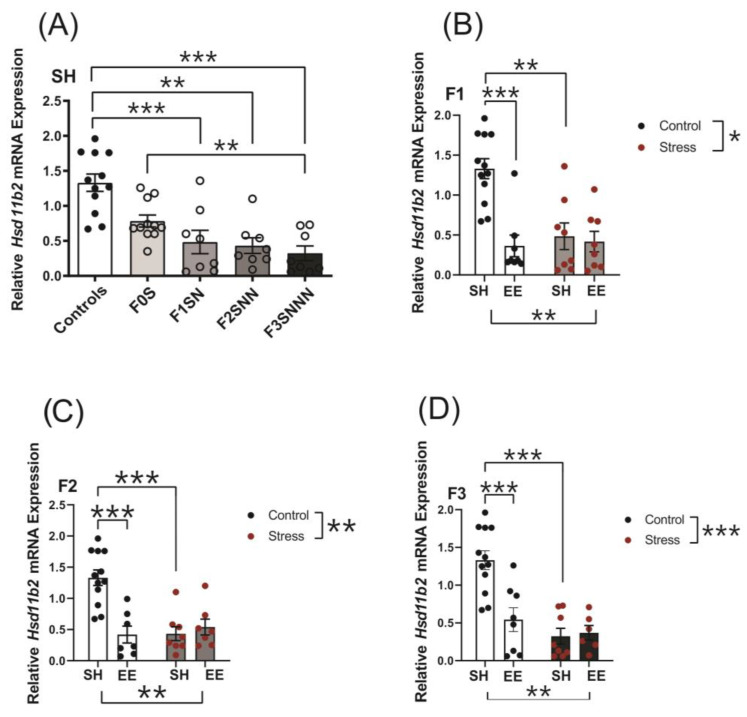
Uterine expression of *Hsd11b2* is significantly decreased in F1–F3 stressed dams and in the animals exposed to EE housing. (**A**) Gene expression of *Hsd11b2* in uteri of control and stressed dams across the F0–F3 generations. Uterine expression of *Hsd11b2* in (**B**) F1, (**C**) F2, and (**D**) F3 animals exposed to different treatments and housing conditions. Asterisks indicate significance: <0.05 (*); 0.002 (**); <0.001 (***). Data are compared to F0N, mean ± SEM. Ordinary one-way ANOVA (**A**) and two-way ANOVA (**B**–**D**) analyses were used. N = 8–12 (**A**,**B**); 7–12 (**C**); or 6–12 (**D**) per group. SH = standard housing; EE = enriched housing; F = filial generation; S = stressed; N = nonstressed.

Uterine gene expression of stress markers involved with GC metabolism, inflammation, and parturition was analyzed. Expression levels of *Hsd11b2* were decreased in the F1–F3 offspring (F (4,42) = 9.376, *p* < 0.001; Figure 10A). Further pairwise comparisons showed significantly decreased expression of *Hsd11b2* in the F1SN (*p* < 0.002), F2SNN (*p* < 0.002), and F3SNNN (*p* < 0.001) generations compared to F0N. A significant interaction was observed between treatment and housing on *Hsd11b2* mRNA expression in the F1–F3 offspring (F (1,32) = 6.808, *p* = 0.014; F (1,30) = 15.050, *p* < 0.001; F (1,30) = 9.624, *p* = 0.004, respectively; Figure 10B–D). The expression of *Hsd11b2* decreased in all the F1 generation groups regardless of treatment and housing conditions (F0N vs. F1NN-EE *p* < 0.001; F0N vs. F1SN-SH *p* = 0.001; Figure 10B). Similarly, we demonstrated a significant drop in *Hsd11b2* expression in the F2NNN-EE and F2SNN-SH groups compared to the controls (*p* < 0.001 for both; Figure 10C). We also observed reductions in the expression of *Hsd11b2* in the F3NNN-EE and F3SNN-SH groups compared to the controls (*p* < 0.001 for both; Figure 10D).

#### 2.6.2. Mineralocorticoid Receptor (*Nr3c2*)

**Figure 11:**
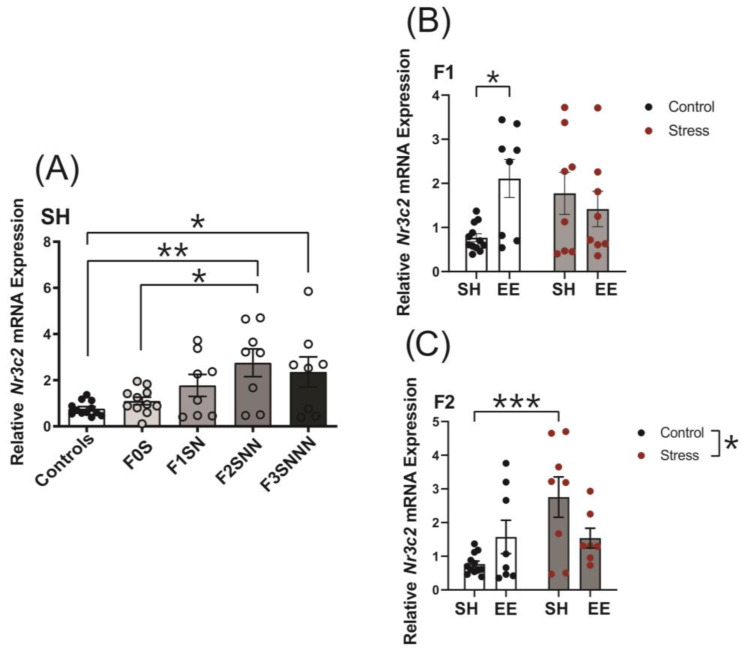
Uterine gene expression analysis of *Nr3c2* showed significant increases in stressed F2 and F3 dams under SH, with similar effects shown when raised under enrichment. (**A**) *Nr3c2* mRNA levels across the F0–F3 generations of stressed dams compared to controls. Analysis of *Nr3c2* expression in the (**B**) F1 and (**C**) F2 generations of dams exposed to different treatments and housing types. Asterisks indicate significance: <0.05 (*); <0.002 (**); <0.001 (***). Data are compared to F0N, mean ± SEM. Ordinary one-way ANOVA (**A**) and two-way ANOVA (**B**,**C**) analyses were used. N = 8–12 (**A**,**B**) or 7–12 (**C**) per group. SH = standard housing; EE = enriched housing; F = filial generation; S = stressed; N = nonstressed.

Uterine expression of *Nr3c2* was upregulated in stressed offspring (F (4,42) = 4.584 *p* = 0.004; Figure 11A), with increased levels in the F2SNN and F3SNNN generations compared to F0N (*p* < 0.002 and *p* < 0.05, respectively) and F0S (F0S vs. F2SNN *p* < 0.05). The analysis of the effects of housing and treatment revealed a significant interaction between factors on *Nr3c2* expression in the F1 and F2 generations (F (1,32) = 6.040, *p* = 0.02 and F (1,31) = 6.915, *p* = 0.013, respectively; Figure 11B,C). Pairwise comparisons showed no significant changes in *Nr3c2* expression between housing conditions in F1 stressed animals (*p* = 0.741), although its expression increased significantly in the F1NN group compared to the controls (*p* = 0.014). We did not observe a main effect of housing on *Nr3c2* expression in the F2 generation (F (1,31) = 0.289, *p* = 0.595), but we did see a significant increase in the F2SNN-SH group compared to the controls (*p* < 0.001; Figure 11C). Two-way ANOVA revealed no significant interaction or main effects of treatment and housing on *Nr3c2* mRNA expression in the F3 uteri (Supplementary Figure S2C).

Gene expression of corticotropin-releasing hormone (*Crh*), corticotropin-releasing hormone receptor 1 (*Crhr1*), corticotropin-releasing hormone receptor 2 (*Crhr2*), *Hsd11b1*, and *Nr3c1* were unchanged in the uteri of stressed animals when compared to the controls (Supplementary Figure S3C–G).

#### 2.6.3. Antioxidant Enzyme Gene Expression: Superoxide Dismutase Type 1 (*Sod1*)

**Figure 12:**
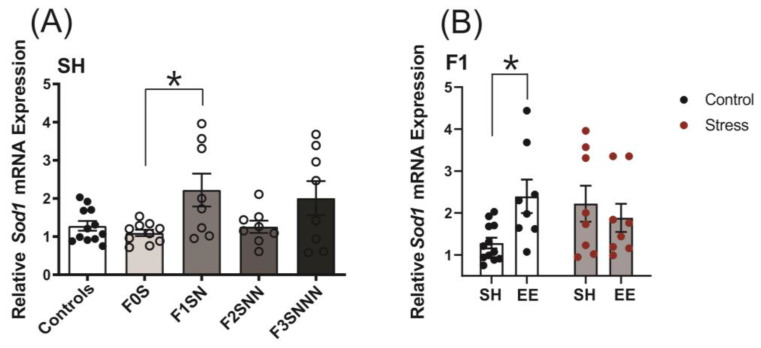
Uterine expression levels of the antioxidant enzyme *Sod1* were significantly increased in F1 stressed animals and in F1 controls subjected to EE conditions. (**A**) Expression of *Sod1* in uteri of stressed F0–F3 dams compared to controls. (**B**) Uterine expression of *Sod1* in F1 females exposed to stress and EE housing compared to controls and SH. Asterisks indicate significance: <0.05 (*). Data are compared to F0N, mean ± SEM. Ordinary one-way ANOVA (**A**) and two-way ANOVA (**B**) analyses were used. N = 8–12 per group. SH = standard housing; EE = enriched housing; F = filial generation; S = stressed; N = nonstressed.

The analysis of antioxidant enzymes in the uteri of rats revealed a significant upregulation of *Sod1* mRNA expression in the F1SN group compared to the controls (F (4,41) = 3.503, *p* = 0.015; Figure 12A). We also observed a significant interaction between treatment and housing conditions on *Sod1* expression in the F1 generation (F (1,32) = 5.289, *p* = 0.028; Figure 12B), but no significant main effects (treatment F (1,32) = 0.459 *p* = 0.503; housing F (1,32) = 1.521, *p* = 0.227). Two-way ANOVA revealed no significant interaction or main effects of treatment and housing on *Sod1* mRNA expression in the F2 and F3 uteri (Supplementary Figure S2D,E). Gene expression of superoxide dismutase type 2 (*Sod2*) was unchanged in the uteri of stressed animals under SH when compared to the controls (Supplementary Figure S3H).

### 2.7. Uterine Concentration of IL-1α, IL-1β, IL-10, IL-6, and TNF-α Cytokines

The concentrations of IL-1α (IL-1α), IL-1β, IL-10, IL-6, and TNF-α were measured in uterine tissue homogenates and the plasma of controls and stressed animals. We did not detect quantifiable amounts of these cytokines in the plasma of the animals. Similarly, IL-6 and TNF-α were not detected in uterine homogenates regardless of the treatment group.

**Figure 13:**
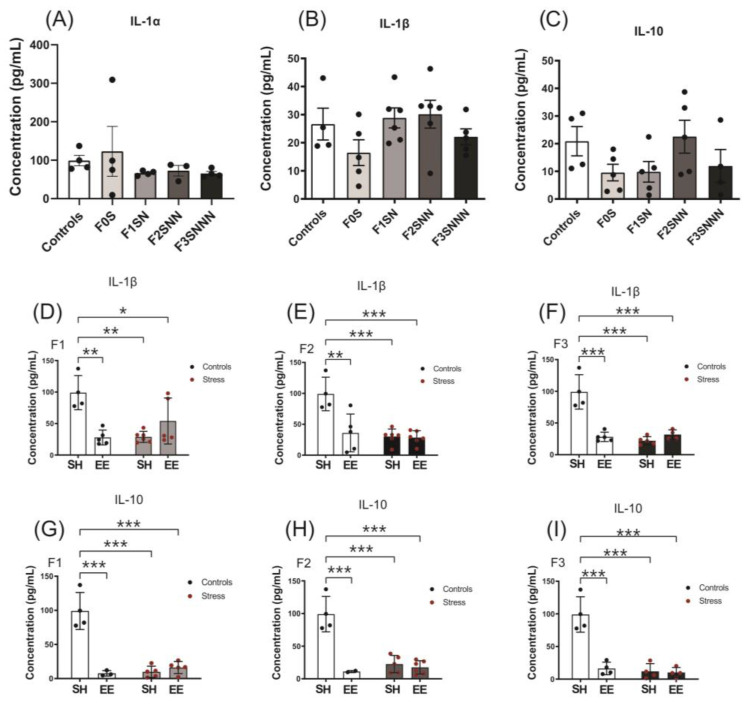
The concentrations of IL-1α, IL-1β, and IL-10 were unchanged between control and stressed groups in uterine tissues. The IL-1β and IL-10 concentrations were increased in the control group compared to animals raised under enriched housing. (**A**–**C**) Uterine concentrations of IL-1α, IL-1β, and IL-10 between control and stressed animals raised under SH. Concentrations of IL-1β (**D**–**F**) and IL-10 (**G**–**I**) in the F1–F3 uteri of dams exposed to different treatments and housing types. Asterisks indicate significance: <0.05 (*); <0.002 (**); <0.001 (***). Data are compared to F0N, mean ± SEM. Ordinary one-way ANOVA (**A**–**C**) and two-way ANOVA (**D**–**I**) analyses were used. N = 3–6 per group. SH = standard housing; EE = enriched housing; F = filial generation; S = stressed; N = nonstressed.

We found no significant changes in IL-1α, IL-1β, and IL-10 concentrations between controls and stressed animals raised under SH (*p* = 0.213, *p* = 0.144, and *p* = 0.216, respectively; Figure 13A–C). However, a significantly higher concentration of IL-1β and IL-10 was observed in the control group compared to stressed-SH, control-EE, and stressed-EE animals in all generations (IL-1β interaction: F1 *p* < 0.001, F2 *p* = 0.004, F3 *p* < 0.001; Figure 13D–F) (IL-10 interaction: F1–F3 *p* < 0.001; Figure 13G–I). There was a significant effect of treatment and housing for IL-1β and IL-10 in all generations (Figure 13D–I), whereas no significant differences were observed for IL-1α in any generation (Supplementary Figure S4A–C).

## 3. Discussion

We provide evidence that psychological and psychosocial chronic variable stress (CVS) alters the inflammatory status and endocrine markers in the uteri of adult dams through transgenerational programming of the female germline over four generations. Our CVS model was insufficient to induce preterm birth or to influence neonatal birth weights. Instead, we observed a boost in resilience to stress in this cohort. EE therapy in prenatally stressed F1 offspring had no beneficial effects on the uterine expression of inflammatory and endocrine markers for them or their future offspring; rather, it generated patterns similar to the stress group.

Both preterm and term labour are inflammatory events characterized by the infiltration of leukocytes into gestational tissues and increased levels of proinflammatory cytokines and chemokines [21,48]. Maternal stress may disrupt this sensitive immunological balance and lead to birth complications (i.e., preterm birth [4,49,50], preeclampsia [51,52]) and inflammation in the placenta and fetal brain [5,53,54]. Restraint and social isolation stressors have been associated with uterine inflammatory, endocrine, and epigenetic modifications in gestational tissues [3,17]. Other consequences of such stressors include metabolic, behavioural, and APOs in rats [43,55,56]. We previously showed that gestational exposure to social isolation stress shortened gestation times in the stressed dams [17], while restraint and forced swimming hastened parturition in the exposed offspring [3]. We proposed that combining two variable stressors would lead to preterm birth in the dams exposed to gestational stress and in their offspring subjected to ancestral stress. Unexpectedly, no changes in pregnancy duration were observed in this study across the F0–F2 generations of stressed rats. These data align with previous maternal stress studies in which gestational stress did not influence pregnancy lengths [28,57].

Ancestral stress increased CORT concentrations in F1 and F2 adult rats, suggesting transgenerational changes in GC programming. These findings align with our prior work, in which multigenerational restraint and forced swimming stressors elevated CORT levels in the F2 offspring [3]. PNMS often intensifies CORT secretion, contributing to APOs and disrupted offspring neurodevelopment [58], but contradictory findings indicate that HPA axis regulation during gestation is complex and related to the stress type.

The dams exposed to CVS had normal pregnancy lengths, and offspring birth weights remained the same, suggesting the animals became more resilient to offset the risk of stress vulnerability on pregnancy and offspring outcomes. This hypothesis is in line with the predictive adaptive response concept, which states that environmental cues acquired in early life build resilience and accommodate future stress hits that may occur later [59,60]. However, the dysregulated HPA axis in the F1 and F2 offspring and altered uterine gene expression of inflammatory and stress markers in response to CVS suggest the opposite. Furthermore, F2 neonates of EE-housed dams were heavier, regardless of the treatment group. Together, these results highlight the complexity of building resiliency in response to stress. It involves multiple physiological systems [61], may be influenced by sex and environmental factors [43,62], and results in different physiological outcomes (e.g., neuroendocrine, behavioural, and immunological responses) [61]. Finally, there is the individual’s susceptibility to different types of stress and the subsequent epigenetic responses and mechanisms [43,63].

Our CVS protocol reduced the uterine mRNA expression of *Il1a* in the F1 and F3 generations, whereas it remained unchanged in the F2 generation. There have been other reports of the transgenerational effects of stress skipping generations and reappearing in future progeny in a sex-specific fashion [17,64], possibly due to epigenetic regulations such as genomic imprinting. Our results are similar to another study in which F1 neonates showed a downregulation in uterine *Il1a* expression after prenatal exposure to a combination of psychological and immunological stressors [28]. It was also reported that multigenerational PNMS in mice elicited immunosuppressive effects in the F2 generation, which were resolved in the F3 animals, suggesting a compensatory mechanism against cumulative stress [4]. We propose that the reduced *Il1a* expression observed here may be a compensatory downregulation initiated by fetal programming of the F1 and F3 offspring in response to adversity.

In contrast to *Il1a* regulation, uterine *Il1b* expression was upregulated in the F2-stressed animals. It is possible that transgenerational CVS increased the allostatic load and exceeded the ability of the F2 generation to cope, resulting in the activation of inflammatory pathways—although not enough to cause preterm birth. These findings are expected given that preterm birth is a complex syndrome that involves early activation of multiple pathways that ultimately trigger labour [21].

We hypothesized that IL-1RAP levels would increase in the uteri of stressed dams, since stress is a potential regulator of IL-1 receptors and accessory proteins [65], and IL-1RAP levels are increased in preeclamptic placentas [66]. However, IL-1RAP protein abundance remained unchanged for all treatments, generations, and housing situations. This finding concurs with a rat study in which IL-1RAP protein abundance was unchanged during labour even though *Il1rap* expression was increased in the upper uterus [65].

We also measured two proinflammatory cytokines (IL-1α and IL-1β) and one anti-inflammatory cytokine (IL-10) in the uterine tissues of animals raised under SH. The multiplex analysis revealed no changes in the concentrations of these cytokines between control and stressed animals in contrast to our gene expression results. Similar findings were previously observed in animals exposed to social isolation stress before and during pregnancy. The uterine concentrations of IL-1α were unchanged due to social isolation stress in the F0 and F1 generations, but IL-1β concentrations were reduced in the F1 offspring of stressed animals [17].

Exposure to CVS induced the downregulation of *Hsd11b2* expression in the F1–F3 offspring’s uteri, suggesting that CORT levels might be higher in their uteri. We also observed increased *Il1b* expression in the F2-stressed uteri, which aligns with previous studies in which proinflammatory cytokines inhibited human placental 11β-HSD2 activity [35]. Still, it opposes our findings on uterine *Hsd11b2* expression in response to social isolation stress [17]. The attenuation of 11β-HSD2 activity by prenatal stressors is linked to preterm birth, low birth weight, and neurodevelopmental delays in the progeny [67,68]. Our CVS protocol did not alter offspring birth weights or pregnancy lengths, but future behaviour and brain development analysis would further clarify its effects on the offspring.

CVS increased uterine expression of the mineralocorticoid receptor (MR), *Nr3c2*, in the F2 and F3 generations. This finding correlates with the reduced expression levels of *Hsd11b2* in these animals since 11β-HSD2 controls CORT binding to MR and strictly regulates the actions of steroid hormones [69]. Although the roles of aldosterone and MR during pregnancy are unclear, they are known to modulate the immune system function, regulate oxidative stress, and release proinflammatory cytokines [70]. We speculate that uterine MR expression modulates inflammatory responses during pregnancy, and stress alters the inflammatory balance. The increased *Nr3c2* and *Il1b* and reduced *Hsd11b2* expression patterns in the uteri of stressed F2 offspring also suggest that this imbalance is passed on transgenerationally.

We used staining with DHE fluorescent probe [71] to assess levels of ROS. Our data demonstrated that ROS levels were unchanged across four generations of transgenerationally stressed rats. Still, we found increased *Sod1* uterine expression in F1-stressed animals, suggesting that a compensatory mechanism was triggered in the F1 generation to counterbalance the effects of ancestral stress experienced in utero. Since SODs are antioxidant enzymes that catalyze the conversion of two superoxide anions to hydrogen peroxide and molecular oxygen [72], these data suggest that SODs regulate the balance of ROS production during pregnancy [73] to prevent damage to cell components [72].

Most of the literature describes enrichment intervention as an optimal housing condition that produces beneficial, albeit variable, effects on neuroanatomical and behavioural assessments [40,44,74,75,76,77,78]. EE has also been used as an attempt to alleviate or reverse transgenerational stress programming [40,44,78,79]. In this study, enrichment intervention resulted in uterine gene expression patterns across the generations that were similar to stressed offspring raised in SH. Similar expression patterns for *Il1a* and *Il1b* proinflammatory cytokines were seen between stressed-SH and stressed-EE animals in the F1 and F2 generations. The transgenerational effects of EE were also observed with increases in *Il1b* expression in F2 controls. The expression patterns of the *Hsd11b2*, *Nr3c2*, and *Sod1* genes in the EE-raised control dams were also similar to stressed offspring. Furthermore, IL-1β and IL-10 concentrations were significantly reduced in the uteri of control and stressed animals subjected to enriched housing and stressed rats raised in SH compared to SH controls. This indicates that CVS induced a downregulation in protein concentration of cytokines in the uteri of enriched rats following the same patterns of stressed-SH animals, as observed in our gene expression analysis. These findings are supported by previous studies in which enrichment interventions promoted stress-like effects [79,80,81,82], anxiety, and reduced social interactions in a sex-specific manner [79].

The sudden change in environment may have been perceived as a threat to the rats, when they were involuntarily and unexpectedly introduced to a novel cage to which they were not acclimated. This hypothesis is supported by previous findings in which birds exposed to short-term EE perceived the introduction of new objects as a stressor [82]. In addition, male rats subjected to 40 days of EE showed signs of chronic stress depicted as altered neuroendocrine regulation by enhanced adrenocortical function and larger adrenals [83]. Although we found no changes in CORT levels in the enriched offspring, EE housing could have been perceived as overenrichment even in a long-term intervention protocol as used in this study, in which the recurrent change in the environment could have prevented the animals from habituating. The animals were introduced to various novel objects, food, and wheels, allowing for physical exercise. Indeed, extensive and forced physical exercise has been associated with heightened CORT levels through HPA axis activation [43,84,85]. However, voluntary physical exercise is a vital component of the beneficial effects of EE [43,86,87]. The stress response triggered by EE indicates a type of positive stress, in which the modifications in uterine markers of stress are similar to the negative PNMS. Still, it might not produce the adverse health consequences of chronic stress [83].

How the enrichment paradigms produce different results among studies is not fully understood. The inconsistent EE results are attributed to each variable analyzed and are context- and stress-dependent, which may produce stress resilience or vulnerability [43,79]. This observation agrees with the enrichment intervention used in this study, which has been shown to reverse neuromorphological, motor, and HPA axis deficits due to ancestral stress [40,44]. In contrast, it induced stress vulnerability in uterine stress markers similar to CVS in the present study. Furthermore, different genotypes, species, and enrichment paradigms (mild or intense) have produced a nongenetic individuality in vulnerability or resilience to PNMS [40,88]. The interindividual traits observed may also originate from complex and hard-to-measure microenvironmental effects [88,89].

The evolutionary rationale for the transgenerational inheritance of environmental effects is to prepare the offspring for anticipated adversity later in life. The transmission of environmental cues possibly occurs through epigenetic inheritance of stress via the gametes, in which the parental phenotypic traits are passed down to the progeny [90]. If the adaptations mismatch the environment and become maladaptive, the inherited traits may lead to pathologies and detrimental modifications that persist through generations [90,91].

A shortcoming of the present study is that uterine tissues were collected at lactational day (LD)21 instead of right after labour for practical reasons and because of the transgenerational design of the study. Uterine expression of inflammatory and stress markers may be regulated differently during labour than as indicated at LD21. The findings of this study, thus, may reflect the uterine transcriptome programmed by maternal and ancestral CVS, which may affect future health and pregnancy outcomes. In addition, analysis of proinflammatory cytokine concentrations and 11β-HSD activities in the uterus and placenta would further elucidate the interactions between these mediators and their contributions to intrauterine inflammation and labour initiation in pregnancies subjected to chronic stressors. Another downside of the current study is the lack of cause-and-effect conclusions, which can be inferred from future studies evaluating the transgenerational effects of prenatal CVS on the germline through epigenomic and metabolomic analyses.

The present results indicate that stressing F0 pregnant rats with CVS conveys transgenerational effects to the offspring, including long-lasting modifications in the inflammatory and endocrine status of the adult uteri. These alterations may produce adaptive or disruptive outcomes in the offspring. This study also demonstrates that enrichment induced adverse rather than beneficial effects on uterine biomarkers of stress over generations of rats. Enrichment can be perceived as stressful depending on the context, duration, and timing of enrichment. In addition, the different measured variables respond differently to each enrichment intervention. Further research should clarify the efficacy of EE housing in ameliorating the effects of CVS on behaviour, proinflammatory, and neuronal factors in the brain. Finding an effective therapy to reduce the effects of prenatal stress and identifying predictive biomarkers of stress that can be translated to humans may improve maternal and child health over multiple generations.

## 4. Materials and Methods

### 4.1. Animals

**Figure 14:**
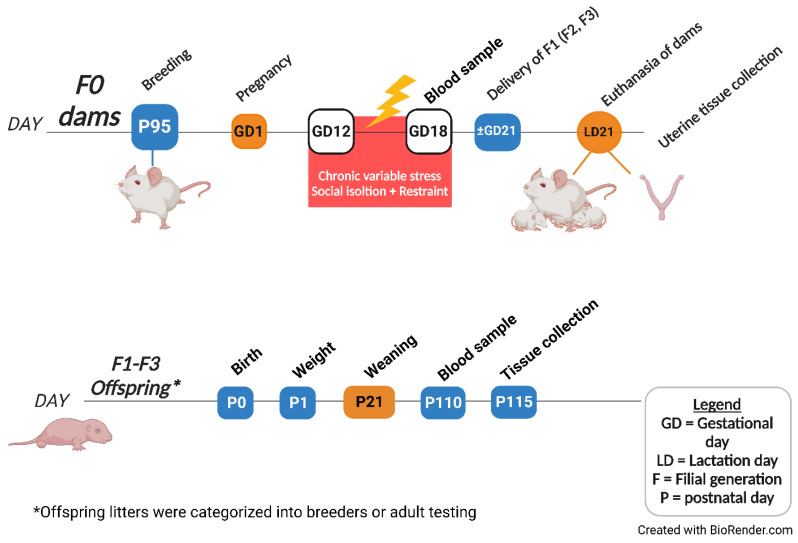
Timeline illustrating the stress protocol, tissue collection, and offspring analyses. Gestational stress was implemented from GD12 to GD18 using restraint and social isolation stressors, creating our psychological and psychosocial chronic variable stress (CVS) model. Blood collection occurred on GD18 in the mothers and on P110 in the offspring. Dams were sacrificed at the weaning of their offspring (LD21) when uterine tissues were collected. Tested offspring were euthanized and had their tissues collected on P115. Created with BioRender.com (accessed on 19 July 2022).

A total of 1495 Long-Evans hooded rats (*Rattus norvegicus*) were used in the colony to produce a maternal (female) prenatal stress lineage of rats. In pairs, nulliparous females were bred and raised at the University of Lethbridge—Canadian Centre for Behavioural Neuroscience vivarium. On postnatal day (P)95, females were individually paired with a stress-free male for one hour a day until successful mating (Figure 14). Pregnancy was confirmed by steady weight gain. Pregnant dams were individually housed from gestational day (GD)20 until delivery, and their gestational hours were video-monitored by continuous infrared light cameras (Panasonic WV-BP330, Panasonic, Minato-ku, Tokyo, Japan) to measure gestational length. Pregnancy duration was measured in hours as the time between the final mounting and delivery of the first pup to precisely capture gestational length changes as previous reported by our group [3]. Maternal data are referred to as GD or lactational days (LD), whereas offspring-related data are described in postnatal days (P). Gestational lengths were monitored, and offspring weights were measured on P1. The pups stayed with their mothers until weaning on P21, followed by housing with same-sex siblings. All experiments were conducted according to the Canadian Council for Animal Care and were approved by the University of Lethbridge Animal Welfare Committee, protocol 1705—Rat Breeding Colony and protocol 1715—Adverse Pregnancy Outcome.

### 4.2. Experimental Design

Three generations of female rats were bred with unstressed control males under standard or enriched conditions, whereby dams and offspring were split according to treatment in each generation (Figure 1). Timed-pregnant females (N = 31–40) from the parental F0 generation were stressed during mid-late gestation (F0S; GD12-18). Their F1 nonstressed female offspring (SN; N = 32–48) were bred to produce the subsequent F2 nonstressed (SNN) generation. The F2SNN female offspring (N = 80–104) were again bred to yield the F3 generation (SNNN; N = 48–56). This cohort generated a transgenerational prenatal stress model of female rats, in which only the F0 pregnant dams were subjected to stress. Each generation’s treatment is depicted by the letters after the filial generation, including stressed rats F0S, F1SN, F2SNN, and F3SNNN and control rats F0N, F1NN, F2NNN, and F3NNNN, in which N depicts nonstressed, and S stressed (Figure 1) [3]. To produce a transgenerational EE lineage, F1 pups were separated into SH or EE housing conditions at weaning (LD21). This produced two lineages of ancestral SH or EE in their F2 and F3 offspring. The animals were kept in their assigned housing condition until GD20, when they were moved to a cage equipped with a camera system to monitor gestational length and maternal behaviour.

### 4.3. Chronic Variable Stress (CVS) Procedures

Timed-pregnant F0 rats underwent periods of restraint and social isolation stress from GD12 to GD18. Stress procedures were implemented at different times and days in a semirandom sequence to avoid habituation to the stressor (see Table 1). For the restraint protocol, animals were placed in a customized transparent plexiglass container for 15–60 min in the morning or evening. The container was placed vertically and adjusted to the animals’ size to prevent them from turning but without compressing their body. The animals were also subjected to 17 h of overnight social isolation stress on GD14 and GD17, in which they were housed alone from 16:00 to 09:00 of the following morning but could still hear and smell their counterparts. The F1–F3 offspring were left unstressed throughout the experiment.

### 4.4. Rearing Environments

The rats were housed under a circadian cycle (12:12 h light/dark cycle) with lights on at 7:30 am, room temperature set at 20 °C, and relative humidity at 30%. The animals had ad libitum access to water and food throughout the experiments. The F1 generation animals were assigned to SH or EE housing conditions from P21 to GD20. For the SH conditions, rats were housed with nonsibling pairs in a standard shoebox-sized plexiglass cage of 8 in. height × 8 in. width × 16 in. depth. They were also offered a standard rodent diet and water ad libitum. Rats assigned to EE were housed in communal condos measuring 24 in. height × 33 in. width × 22 in. depth. They were housed with 4–5 counterparts and were given a standard rodent diet and novel types of food, toys/wheel, and shelters that were changed weekly.

### 4.5. Tissue Collection

#### 4.5.1. Uterine Tissue

Dams were euthanized with euthanyl (sodium pentobarbital) 300 mg/kg (Cambridge, ON, Canada) anesthesia on LD21 or P115. Uterine horns were dissected and snap-frozen for mRNA and protein analyses (N = 6–12 and N = 3–4, respectively). Snap-frozen tissues were placed at –20 °C prior to tissue embedding. Pieces of uterine horns were embedded in optimal cutting temperature medium compound (Tissue-Tek^®^ OCT, Sakura Finetek, CA, USA) and snap-frozen for subsequent analysis. Tissues embedded in OCT were cut at 5 μm, mounted at –20 °C, and stored at –80 °C until use.

#### 4.5.2. Blood

Animals under 4% isoflurane (Fressenious Kabi Canada Ltd., Toronto, ON, Canada) had blood samples (0.5 mL) collected from the lateral tail vein between 9:00 and 10:00 am on GD18 for dams and P110 for tested offspring. Plasma was isolated by centrifuging the blood at 5000 rpm for 10 min and then stored at −80 °C until further analyses.

### 4.6. Molecular Analysis

#### 4.6.1. RNA Extraction

Total RNA from F0–F3 uterine horns was extracted using Trizol (Thermo Fisher Scientific, Wilmington, DE, USA) and Qiagen RNeasy Mini Kit on QIAcube (Qiagen, Toronto, ON, Canada) following the manufacturer’s protocol. A NanoDrop ND-1000 spectrophotometer (Thermo Fisher Scientific, Wilmington, DE, USA) was used to quantify the total RNA. A 260/280 nm absorbance ratio of ~2.0 was considered pure.

#### 4.6.2. Quantitative Real-Time Polymerase Chain Reaction (RT–qPCR)

RT–qPCR was used to quantify genes involved in parturition, inflammation, and stress-related pathways in the uterine horns. The genes selected were inflammatory markers *Il1a*, *Il1b*, *Il1ra*, and *Il6* and stress markers *Crh*, *Crhr1*, *Crhr2*, *Hsd11b1*, *Hsd11b2*, glucocorticoid receptor *(Nr3c1)*, *Nr3c2*, *Sod1*, and *Sod2*.

The reverse transcriptase reaction was performed with total RNA (500 ng) to produce complementary deoxyribonucleic acid (cDNA) using iScript Reverse Transcription Supermix (Bio-Rad Laboratories, Mississauga, ON, Canada) according to the manufacturer’s protocol. The primers used in these studies were previously designed by our group, assuring that the 3′ and 5′ primers spanned over an exon–exon boundary to avoid primers binding to genomic deoxyribonucleic acid (DNA). Primer sequences, annealing temperatures, and accession numbers are described in Table 2 primers. The PCR was completed in duplicate by adding 0.5 μL forward and 0.5 μL reverse primer (10 μM), 10 μL iQ SYBR Green Supermix (Bio-Rad Laboratories, Mississauga, ON, Canada) and 9 μL of cDNA (25 ng/μL) for a total reaction of 20 μL/well. Two-step quantitative RT-PCR (amplification and melt curve analysis of nonspecific products) with denaturation at 95 °C for 10 min, annealing and elongation for 15 s at 95 °C, and 1 min at the primer-specific annealing temperature (Table 2 primers) were run in iCycler iQ thermal cyclers (Bio-Rad Laboratories, Mississauga, ON, Canada). A pooled sample was prepared with three different cDNA samples combined to assess batch-to-batch repeatability between the same gene experiments. The pooled sample was included in all PCR plate analyses with proper threshold cycle (Ct) adjustments prior to data analysis.

Data analyses were conducted as previously described by Leimert et al. [92]. In brief, cDNA samples were serially diluted to produce a standard curve for each PCR reaction (target genes and the housekeeping gene *Ppia*) and analyzed with iCycler IQ software (Bio-Rad Laboratories, Mississauga, ON, Canada). The equation E = 10^−1/slope^ was used to determine the reaction amplification efficiency using the slope of the standard curve. The average Ct value for each sample was corrected by the efficiency of the reaction. This was repeated for all genes selected in this study. The final threshold cycles were expressed relative to the pooled sample. Target genes data were analyzed according to the Pfaffl method [93] relative to Cyclophilin A (Peptidilprolyl Isomerase A or *Ppia*) gene expression using the formula:

$$Expression\;ratio=\frac{E_{Target}{}^{\Delta Ct\left(Control-Sample\right)}}{E_{Ref}{}^{\Delta Ct\left(Control-Sample\right)}}$$

### 4.7. Western Blot

Pieces of snap-frozen uterine horns were homogenized using a Qiagen TissueLyser II (2 min at 25 Hz, 3 times; Qiagen, Toronto, ON, Canada) in radio immunoprecipitation assay (RIPA) buffer (1 M Tris pH 8, 5 M sodium chloride, 500 mM ethylenediaminetetraacetic acid (EDTA) pH 8, Triton X-100, 200 mM phenyl methane sulfonyl fluoride (PMSF)) containing freshly added HALT protease inhibitor cocktail (100x; Thermo Fisher Scientific, Wilmington, DE, USA). Protein lysate was stored at –80 °C until Western blot analysis. The protein concentration of samples was determined using a NanoDrop ND-1000 spectrophotometer (Thermo Fisher Scientific, Wilmington, DE, USA) with Precision red advanced protein assay reagent (Cytoskeleton Inc., Denver, CO, USA). The total protein for each sample (20 μg) was combined with 1x loading buffer (250 mM Tris-hydrochloric acid (HCl) containing 4% sodium dodecyl sulphate (SDS), 10% glycerol, 2% β-mercaptoethanol, and 0.002% bromophenol blue) and denatured at 95 °C for 5 min. The protein lysates were separated by SDS (12% *w*/*v*)-polyacrylamide gel electrophoresis (SDS-PAGE) and transferred to nitrocellulose membranes by electroblotting. Membranes were incubated with intercept TBS blocking buffer (LI-COR Biosciences, Lincoln, NE, USA) for 1 h at room temperature and were subsequently incubated with the primary antibodies anti-IL1RAP at 1:1000 (66 kDa, Abcam, ab8110; Cambridge, UK) and anti-glyceraldehyde 3-phosphate dehydrogenase (GAPDH) at 1:5000 (37 kDa, Thermo Fisher Scientific, PA1-987; Wilmington, DE, USA) overnight at 4 °C. Membranes were then washed 3 times with phosphate-buffered saline (PBS) containing 0.1% Tween 20 (Sigma-Aldrich, San Louis, MO, USA) and incubated with secondary IRDye 800CW antibody (LI-COR Biosciences, Lincoln, NE, USA) at 1:5000 at room temperature. The intensities of the light-emitting bands were quantified using the Odyssey LI-COR Biosciences Infrared Imaging System and application software V3.0 (LI-COR Biosciences, Lincoln, NE, USA). The relative levels of IL1RAP were normalized to GAPDH band intensities, and a ratio of the relative values to an internal blot control was obtained. Data were expressed relative to F0 controls (F0N). Full blot images are presented in the Supplementary Figure S5.

### 4.8. Superoxide Detection Assay

Intracellular superoxide levels were measured by staining with 25 μM of dihydroethidium (DHE 25 mg; Biotium Inc., Fremont, CA, USA) to detect oxidative stress in uterine horns. Uterine tissues were washed with Hank’s Balanced Salt Solution (Gibco™ HBSS, calcium, magnesium; Thermofisher, ON, Canada) and incubated for 10 min at 37 °C in a humid chamber. Subsequently, 25 μM of DHE was added and incubated for 30 min at 37 °C. Excess DHE was washed away after the incubation time, and the sample was quickly cover-slipped and imaged. Images were captured using a fluorescence microscope (IX81; Olympus, Tokyo, Japan) with a CoolSNAP HQ2CCD camera (Photometrics, Huntington Beach, CA, USA) using cellSens Dimensions, version 1.9 (Olympus, Tokyo, Japan) with TRITC at 532 nm wavelength. Images were analyzed with Fiji ImageJ (National Institutes of Health, Bethesda, MD, USA) to assess mean fluorescence intensity (MFI). Duplicate images were taken for each sample from 4 regions (i.e., top, bottom, left, and right) at 20× magnification. All images were corrected to background fluorescence, and their respective MFIs were averaged and normalized to the average of the F0 control animals.

### 4.9. Luminex Cytokine Assays

Cytokine levels for IL-1α, IL-1β, IL-6, IL-10, and TNF-α from the F0–F3 generations were quantified simultaneously. Analyses were performed using Bio-Plex 200 suspension array system and Bio-Plex 200 software, version 6.0 (Bio-Rad Laboratories, Mississauga, ON, Canada). We used the MILLIPLEX MAP Rat Cytokine/Chemokine Magnetic Bead Panel (RECYMAG-65K), a precustomized magnetic-bead-based multiplex assay (Millipore Sigma, Burlington, MA, USA) and followed the manufacturer’s protocol. In brief, uterine horns (3 mm) were weighted and diluted with 1x phosphate-buffered saline (PBS) to a concentration of 0.1 mg/mL. Tissues were homogenized using Tissue Lyzer II (Qiagen, Toronto, ON, Canada) with 7 mm stainless steel beads 4 times for 2 min, 25 Hz cycles. Tissue homogenate protein concentrations were quantified using a NanoDrop ND-1000 spectrophotometer (Thermo Fisher Scientific, Wilmington, DE, USA) with Precision red advanced protein assay reagent (Cytoskeleton Inc., Denver, CO, USA), and then immediately stored at −80 °C until use. Multiplex assay was calibrated and validated before sample analyses. Reagents’ preparation and assay were conducted following the manufacturer’s protocol.

### 4.10. Statistical Analyses

All statistical analyses were performed using GraphPad Prism (version 5.0 and 9.0; GraphPad Prism, La Jolla, CA, USA). Data were tested for normal distribution and log10-transformed when necessary prior to statistical testing. One-way analysis of variance (ANOVA) test was used, followed by Tukey’s post hoc test when significance was achieved (*p* < 0.05). The nonparametric Kruskal-Wallis test was used when the data did not follow the assumptions of parametric one-way ANOVA, as is indicated in the figure legends, followed by Šidák’s post hoc tests for multiple comparisons when significance was achieved. The interaction between treatment and housing for each generation was measured using two-way ANOVA, and significant results were explored using Tukey’s tests for multiple comparisons. Results are expressed as mean ± SEM. Box plot mid-lines indicate medians, whiskers indicate min-max values, and boxes indicate interquartile ranges.

## Figures and Tables

**Table 1:** Description of stress procedures implemented through GD12-18 using restraint and social isolation. N/A describes periods when the rodents were not stressed.

Gestational Day	G-12	G-13	G-14	G-15	G-16	G-17	G-18
**Treatment (am)**	Restraint **60 min**	N/A	Restraint **45 min**	N/A	Restraint **15 min**	N/A	Restraint **45 min**
**Treatment (pm)**	N/A	Restraint **30 min**	Isolation **Overnight**	Restraint **60 min**	N/A	Restraint **30 min**	N/A
						Isolation **Overnight**	

**Table 2:** Primer forward and reverse sequences and annealing temperatures for RT–qPCR.

Target Gene	Forward Primer (5′ → 3′)	Reverse Primer (5′ → 3′)	Annealing Temperature (°C)	NCBI Reference Sequences *
***Ppia (Cyclophilin A)***	CAC CGT GTT CTT CGA CAT CAC	CCA GTG CTC AGA GCT CGA AAG	60	NM_017101.1
***Il1a***	AAGACAAGCCTGTGTTGCTGAAGG	TCCCAGAAGAAAATGAGGTCGGTC	55	NM_017019.1
***Il1b***	CTCAATGGACAGAACATAAGCC	GGTGTGCCGTCTTTCATCA	51	NM_031512.2
***Il6***	TCCTACCCCAACTTCCAATGCTC	TTGGATGGTXTTGGTCCTTAGCC	65	NM_012589.2
***Il1ra***	AAGACCTTCTACCTGAGGAACAACC	GCCCAAGAACACATTCCGAAAGTC	55	NM_022194.2
***Il1rap***	GGGCAACATCAACGTCATTTTAG	CAGCTCTTTCACCTTCAAGTCCTT	68	NM_012968.1
***Hsd11b1***	GAAGAAGCATGGAGGTCAAC	GCAATCAGAGGTTGGGTCAT	60	NM_017080.2
***Hsd11b2***	CGTCACTCAAGGGGACGTAT	AGGGGTATGGCATGTCTCC	55	NM_017081.2
***Crh***	ATCTCACCTTCCACCTTCTG	GTGTGCTAAATGCAGAATCG	60	NM_031019.1
***Crhr1***	GGTGACAGCCGCCTACAATT	AAGGTACACCCCAGCCAA	60	NM_030999.4
***Crhr2***	TGGTGCATACCCTGCCCTAT	GTGGAGGCTCGCAGTTTTGT	60	NM_022714.1
***Nr3c1***	TGTATCCCACAGACCAAAGCA	AATCCTCATTCGTGTTCCCTTC	52	NM_012576.2
***Nr3c2***	GGCAAACAGATGATCCAGG	CAACTCAAAGCGAACGATGA	60	NM_013131.1
***Sod1***	GCAGAAGGCAAGCGGTGA	GGTACAGCCTTGTGTATTGTC CC	60	NM_017050.1
***Sod2***	GTCTGTGGGAGTCCAAGGTT	GTTCCTTGCAGTGGGTCCTGATTA	60	NM_017051.2

* NCBI—National Center for Biotechnology Information (http://www.ncbi.nlm.nih.gov; accessed on 20 November 2022).

## Data Availability

The data presented in this study are available in the article.

## Supplementary Materials

The following supporting information can be downloaded at: https://www.mdpi.com/article/10.3390/ijms24043734/s1.

## Abbreviations

11β-hydroxysteroid dehydrogenase type 1	11β-HSD1
11β-hydroxysteroid dehydrogenase type 2	11β-HSD2
Adverse pregnancy outcomes	APOs
Analysis of variance	ANOVA
Chronic variable stress	CVS
Complementary deoxyribonucleic acid	cDNA
Corticotropin releasing hormone	*Crh*
Corticotropin releasing hormone receptor 1	*Crhr1*
Corticotropin releasing hormone receptor 2	*Crhr2*
Cortisol/corticosterone	CORT
Cyclophilin A	Peptidilprolyl Isomerase A or Ppia
Dihydroethidium	DHE
Enriched environment	EE
Filial	F
Genomic deoxyribonucleic acid	DNA
Gestational day	GD
Glucocorticoid receptor	*Nr3c1*
Glucocorticoids	GCs
Glyceraldehyde 3-phosphate dehydrogenase	GAPDH
Hypothalamic-pituitary-adrenal	HPA
Interleukin (IL)-1 receptor 1	IL-1R1
Interleukin (IL)-1 receptor accessory protein	IL-1RAP
Interleukin (IL)-10	IL-10
Interleukin (IL)-1α	IL-1α
Interleukin (IL)-1β	IL-1β
Interleukin (IL)-6	IL-6
Lactational day	LD
Mean fluorescence intensity	MFI
Mineralocorticoid receptor	*Nr3c2*
Phosphate-buffered saline	PBS
Postnatal day	P
Prenatal maternal stress	PNMS
Quantitative Real-Time Polymerase Chain Reaction	RT–qPCR
Radio immunoprecipitation assay	RIPA
Reactive oxygen species	ROS
Standard housing	SH
Superoxide dismutase type 1	*Sod1*
Superoxide dismutase type 2	*Sod2*
Tumour necrosis factor-alpha	TNF-α
