# Supplementary material for: Environmental Enrichment Promotes Transgenerational Programming of Uterine Inflammatory and Stress Markers Comparable to Gestational Chronic Variable Stress
Source: Int J Mol Sci. 2023 Feb 13;24(4):3734. doi: 10.3390/ijms24043734 (PMC9962069; doi:10.3390/ijms24043734)
Supplement: Supplementary file 1 [file ijms-24-03734-s001.zip › ijms-2069108-supplementary.pdf]

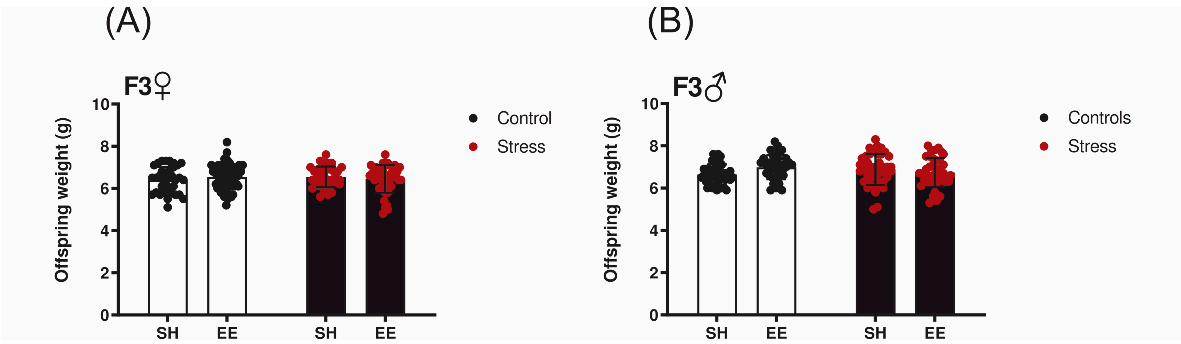

Figure S1: Offspring weights among females (A) and males (B) in the F3 generation.

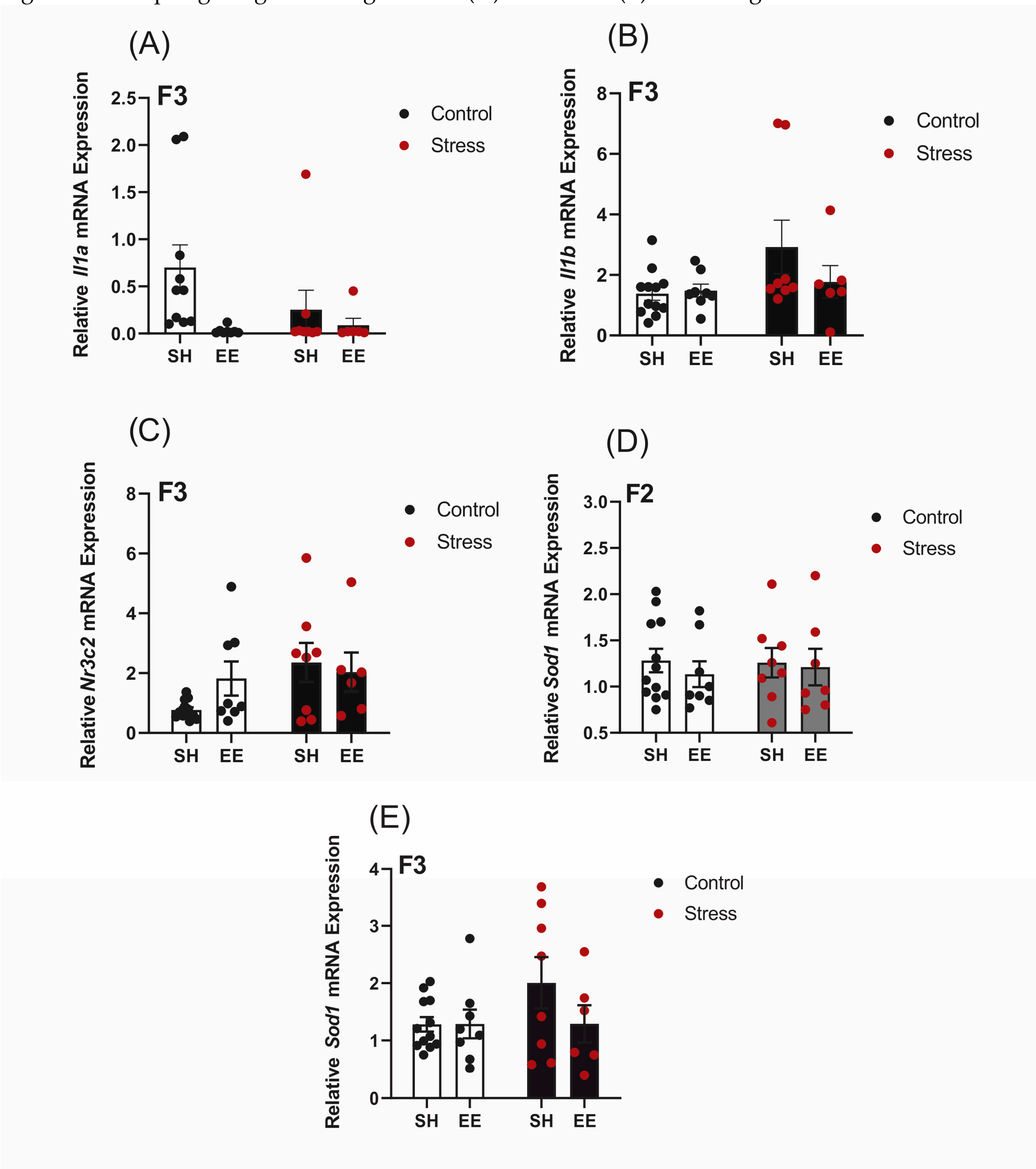

Figure S2: The effects of housing and treatment on inflammatory and stress genes in the F2 (D) and F3 (A–C,E) generations.

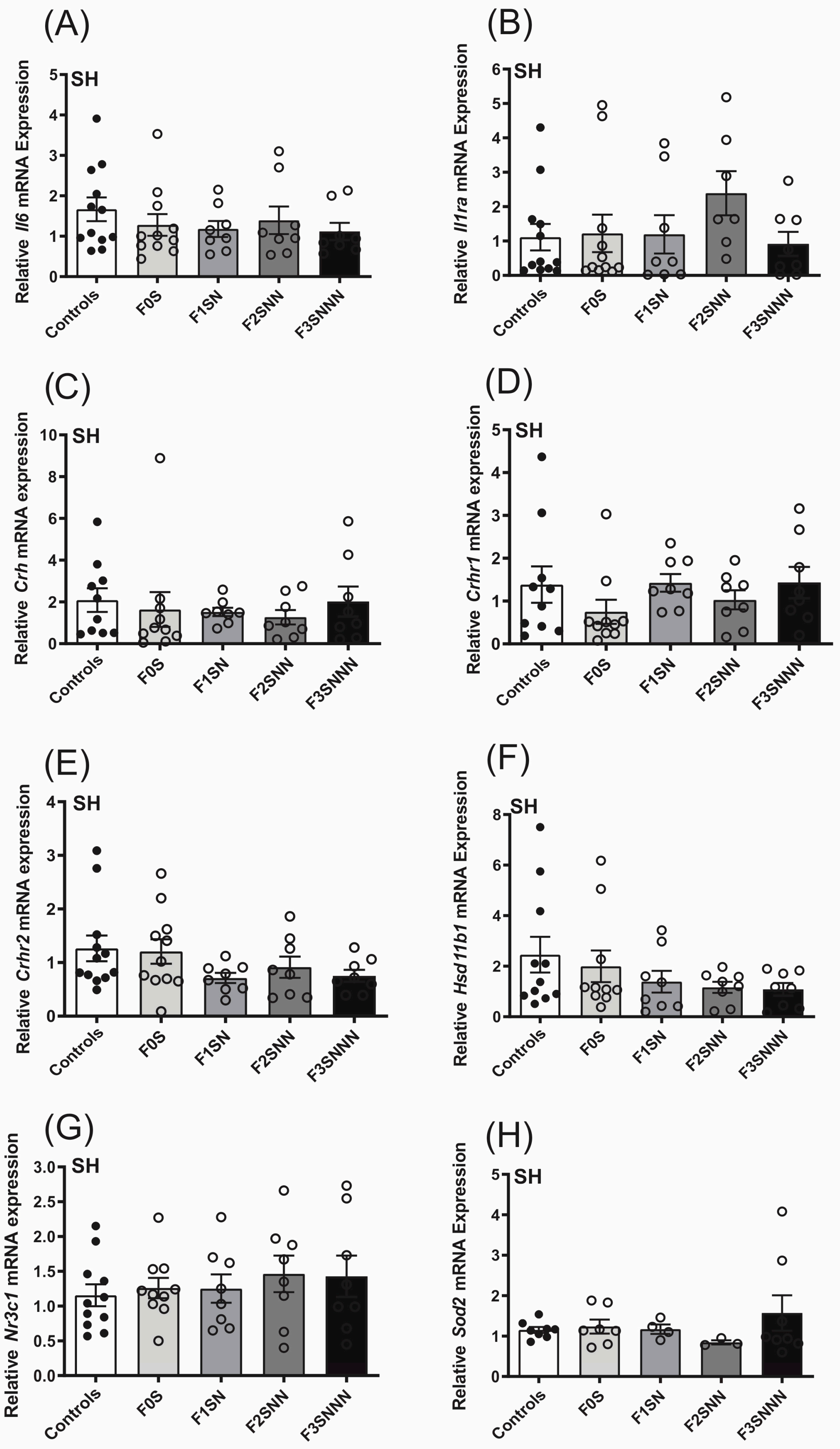
(A)
SH
Relative Il6 mRNA Expression
Controls
F0S
F1SN
F2SNN
F3SNNN
(B)
SH
Relative Il1ra mRNA Expression
(C)
SH
Relative Crh mRNA expression
(D)
SH
Relative Crhr1 mRNA expression
(E)
SH
Relative Crhr2 mRNA expression
(F)
SH
Relative Hsd11b1 mRNA Expression
(G)
SH
Relative Nr3c1 mRNA expression
(H)
SH
Relative Sod2 mRNA Expression

Figure S3: (A–H) CVS effects on inflammatory and stress mediators across generations of rats raised under standard housing.

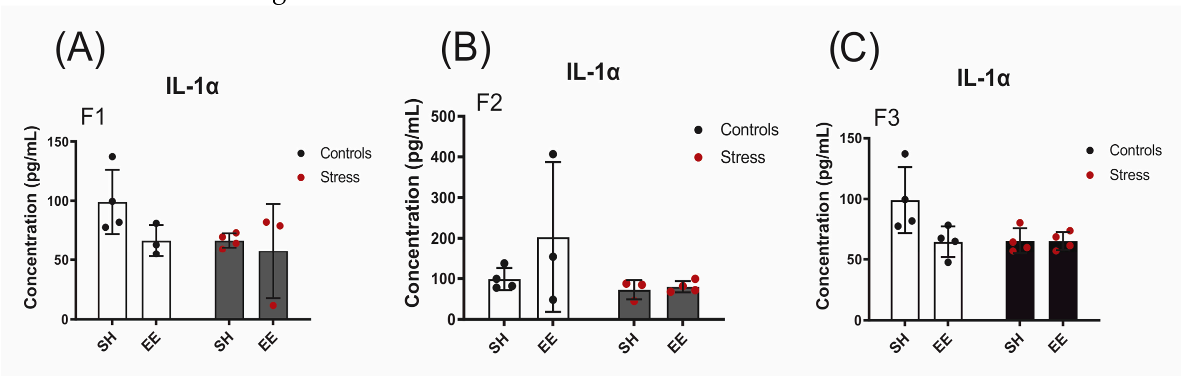

Figure S4: (A–C) The effects of housing and treatment on IL-1α concentrations across the F1-F3 generations of stressed rats.

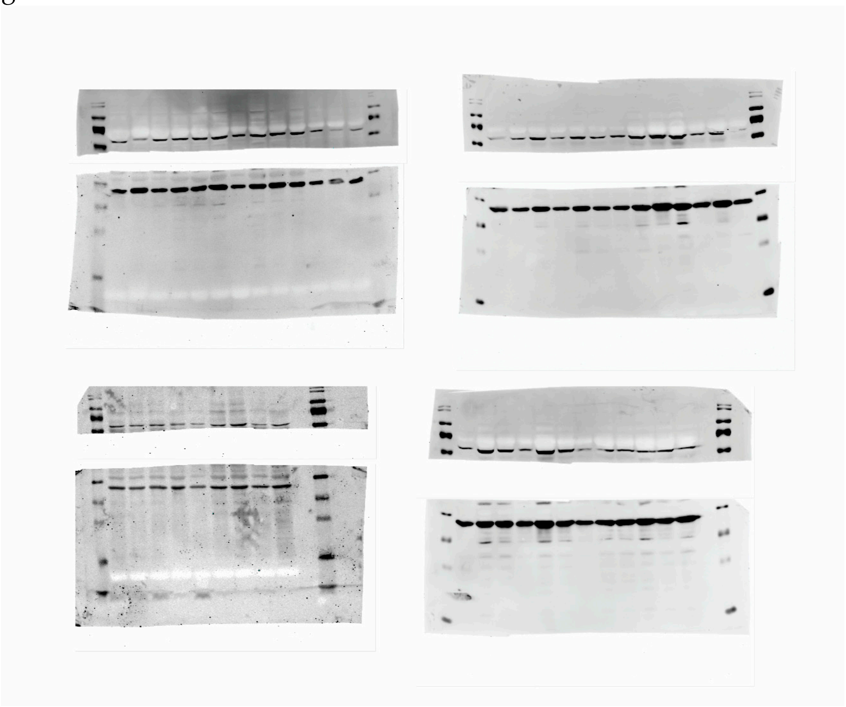

Figure S5: Full blot images of Western Blot analysis.
